# Glucagon-Like Peptide-1 Receptor Agonists and Prior Major Adverse Limb Events in Patients With Diabetes

**DOI:** 10.1001/jamanetworkopen.2025.55952

**Published:** 2026-01-28

**Authors:** Fu-Chih Hsiao, Tzyy-Jer Hsu, Yu-Jui Hsieh, Ying-Chang Tung, Dong-Yi Chen, Chia-Pin Lin, Shao-Wei Chen, Pao-Hsien Chu

**Affiliations:** 1 The Cardiovascular Department, Chang Gung Memorial Hospital, Linkou, Taoyuan, Taiwan; 2 College of Medicine, Chang Gung University, Taoyuan, Taiwan; 3 Division of Thoracic and Cardiovascular Surgery, Department of Surgery, Chang Gung Memorial Hospital, Linkou Medical Center, Chang Gung University, Taoyuan City, Taiwan; 4 Center for Big Data Analytics and Statistics, Chang Gung Memorial Hospital, Taoyuan, Taiwan; 5 Institute of Stem Cell and Translational Cancer Research, Chang Gung Memorial Hospital, Linkou, Taoyuan, Taiwan

## Abstract

**Importance:**

Patients with diabetes and a history of major adverse limb events (MALEs) are at an increased risk of cardiovascular and limb-related complications; however, effective glucose-lowering therapies for secondary prevention in this population are limited.

**Objective:**

To evaluate whether glucagon-like peptide-1 receptor agonists (GLP-1 RAs) are associated with reduced risk of MALEs and major adverse cardiovascular events (MACE) compared with dipeptidyl peptidase-4 (DPP-4) inhibitors in patients with diabetes and prior MALEs.

**Design, Setting, and Participants:**

This retrospective, nationwide cohort study used data from the Taiwan National Health Insurance Research Database from October 2012 to December 2023. Patients with diabetes and a history of MALE who initiated GLP-1 RAs or DPP-4 inhibitors were included. MALEs were defined as chronic limb-threatening ischemia, lower limb revascularization, or nontraumatic minor and major amputation.

**Exposures:**

Initiation of GLP-1 RAs (liraglutide, dulaglutide, or semaglutide) vs DPP-4 inhibitors.

**Main Outcomes and Measures:**

The primary outcome was a composite of lower limb revascularization and nontraumatic major and minor amputation. The secondary outcomes were MACEs (cardiovascular death, ischemic stroke, and myocardial infarction), all-cause mortality, and progression to long-term dialysis. A new-user, active-comparator design with inverse probability of treatment weighting was employed.

**Results:**

Among 17 288 patients (mean [SD] age, 70.7 [12.0] years; 10 010 male [57.9%]), 1583 initiated GLP-1 RAs and 15 705 initiated DPP-4 inhibitors. After weighting, the use of GLP-1 RAs was associated with a lower risk of MALEs (subdistribution hazard ratio [SHR], 0.90; 95% CI, 0.83-0.97), primarily due to a marked reduction in amputation (SHR, 0.86; 95% CI, 0.75-0.98). GLP-1 RAs were also associated with reduced risks of MACEs (HR, 0.62; 95% CI, 0.58-0.65), cardiovascular death (HR, 0.57; 95% CI, 0.53-0.61), all-cause mortality (HR 0.63; 95% CI, 0.60-0.66), and progression to dialysis (SHR, 0.61; 95% CI, 0.54-0.70).

**Conclusions and Relevance:**

In this nationwide cohort study of patients with diabetes and prior MALEs, treatment with GLP-1 RAs was associated with significantly lower risks of recurrent limb events, cardiovascular events, all-cause mortality, and kidney disease progression compared with DPP-4 inhibitors. These findings support the preferential use of GLP-1 RAs for secondary prevention in this high-risk population.

## Key Points

**Question:**

Are glucagon-like peptide-1 receptor agonists (GLP-1 RAs) associated with improved limb, cardiovascular, and kidney outcomes compared with dipeptidyl peptidase-4 (DPP-4) inhibitors in patients with diabetes and prior major adverse limb events?

**Findings:**

In this cohort study of 17 288 patients, the use of GLP-1 RAs was associated with significantly lower risks of limb events, amputation, cardiovascular events, progression to dialysis, and all-cause mortality compared with DPP-4 inhibitors.

**Meaning:**

These findings suggest that GLP-1 RAs may offer important protective benefits for high-risk patients with diabetes and prior limb events, supporting their preferential use in secondary prevention.

## Teaser

This cohort study analyzes associations of glucagon-like peptide-1 receptor agonists (GLP-1 RAs) with risk of recurrent major adverse limb events in patients with diabetes.

## Introduction

Major adverse limb events (MALEs) are caused by severe limb ischemia leading to interventions including major vascular amputation, and they are associated with increased risks of subsequent amputation, myocardial infarction (MI), stroke, and death.^1,2,3,4^ Known risk factors for MALEs include diabetes, hypertension, dyslipidemia, smoking, chronic kidney disease, heart failure, prior major adverse cardiovascular events (MACEs), and previous revascularization procedures.^5,6,7^ The incidence and prevalence of MALEs continue to rise along with the increase in global prevalence of these risk factors, imposing a substantial burden on health care systems worldwide.^8,9,10^

In patients with peripheral arterial disease and type 2 diabetes, the 2024 American College of Cardiology and the American Heart Association guidelines for the management of lower extremity peripheral artery disease recommend the use of glucagon-like peptide-1 receptor agonists (GLP-1 RAs) and sodium-glucose cotransporter 2 inhibitors to reduce the risk of MACEs.^11^ Currently, the combination of low-dose rivaroxaban and aspirin is the only treatment regimen shown to be effective in reducing the risk of subsequent MALEs in patients undergoing revascularization for lower extremity arterial disease (LEAD), regardless of diabetic status.^12^ There remains an unmet need for medical treatments aimed at reducing subsequent cardiovascular risks in patients who have experienced a MALE.

Several observational studies have reported that the use of GLP-1 RAs was associated with a significantly reduced risk of MALEs compared with other glucose-lowering therapies in patients with diabetes. However, these studies primarily involved participants with a low baseline risk for MALEs, given that few had established LEAD or a history of MALEs,^13^ and data remain limited regarding the associations of GLP-1 RAs with limb outcomes among patients with previous MALEs. Therefore, we hypothesized that, among patients with diabetes and a history of MALEs, treatment with GLP-1 RAs would be associated with a lower risk of recurrent MALEs and MACEs compared with other glucose-lowering therapies.

## Methods

### Data Source

This retrospective cohort study used data from the Taiwan National Health Insurance Research Database (NHIRD), a robust, population-based platform maintained by the Health and Welfare Data Science Center. The NHIRD captures comprehensive claims from Taiwan’s mandatory, single-payer National Health Insurance Program. The database covers information from January 1, 2000, to December 31, 2021, including patient demographics, diagnostic codes, hospitalizations, and prescriptions. Diagnoses were coded using the *International Classification of Diseases, Ninth Revision (ICD-9) *prior to 2016 and both the *ICD-9* and *International Statistical Classification of Diseases and Related Health Problems, Tenth Revision (ICD-10)* thereafter. Further details on the NHIRD have been previously described.^14,15,16^ This study adheres to Strengthening the Reporting of Observational Studies in Epidemiology (STROBE) reporting guideline for cohort studies. The protocol was approved by the institutional review board of the Chang Gung Medical Foundation, Taiwan, and conducted according to the Declaration of Helsinki.^17^ Because all personal data in the NHIRD are deidentified through encryption, the requirement for informed consent was waived.

### Study Design

This study was designed as a new-user, active-comparator design to minimize potential biases.^18^ Patients with type 2 diabetes who had a history of MALEs and initiated treatment with either GLP-1 RAs (ie, liraglutide, dulaglutide, and semaglutide) or dipeptidyl peptidase-4 (DPP-4) inhibitors between October 1, 2012, and December 31, 2023, were identified from the NHIRD. DPP-4 inhibitors (ie, sitagliptin, saxagliptin, linagliptin, alogliptin, and vildagliptin) were selected as the comparator group because of their relatively neutral cardiovascular effects reported in previous studies.^19,20^ During the study period, reimbursement for both drugs was restricted to type 2 diabetes. Inclusion required at least 3 consecutive months of medication use. A history of MALEs was defined as the occurrence of any of the following events: (1) chronic limb-threatening ischemia (CLTI); (2) revascularization procedures for LEAD, including endovascular therapy and bypass surgery; and (3) nontraumatic limb major and minor amputation. CLTI was characterized by ischemic rest pain or threatened tissue viability, corresponding to Fontaine stage III or IV. CLTI was identified by at least 2 outpatient diagnoses or 1 inpatient admission, while revascularization and amputation were ascertained via specific inpatient procedural codes.

**Figure 1.**
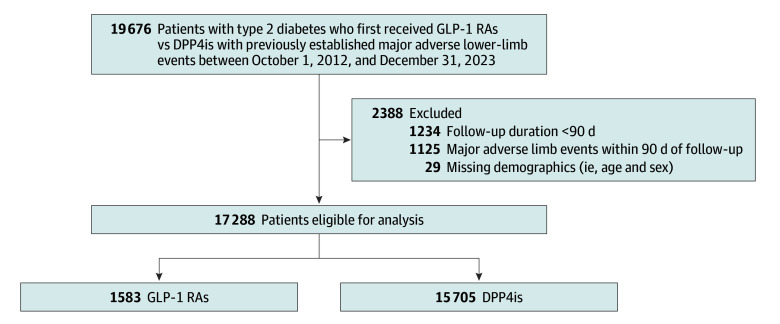
Study Flow Diagram DDP4is indicates dipeptidyl peptidase-4 inhibitors; GLP-1 RAs, glucagon-like peptide-1 receptor agonists.

We excluded patients who had missing demographics, were aged younger than 18 years, had follow-up for less than 90 days, or had a MALE occurrence within 90 days of the index date. Patients were classified into GLP-1 RA or DPP-4 inhibitor groups using outpatient and pharmacy claims (Figure 1); switching within the same drug class was permitted. Follow-up began on the index date (first prescription) and was censored at the earliest of outcome occurrence, treatment switch (between GLP1-RAs and DPP-4 inhibitors), death, or December 31, 2023.

### Outcomes

The primary outcome was composite MALEs (lower limb revascularization or nontraumatic major or minor amputation). Secondary outcomes included MACEs (cardiovascular death, ischemic stroke, and acute MI), all-cause mortality, and new-onset long-term dialysis. Mortality data were linked to the Taiwan National Death Registry. Cardiovascular death comprised acute MI, sudden cardiac death, heart failure, stroke, cardiovascular procedures, cardiovascular hemorrhage, and related causes. Ischemic stroke and MI were identified via principal discharge diagnoses as validated in previous NHIRD studies.^21,22^ New onset of long-term dialysis was determined by the issuance of a Catastrophic Illness Certificate.

### Covariates

A comprehensive list of covariates was considered and classified into 4 main categories: demographics, types of LEAD, comorbidities, event history, and medications. Demographic variables included age, sex, duration of diabetes (which could be traced back to 2000), and diabetic complications such as retinopathy and neuropathy. Comorbidities included hypertension, dyslipidemia, coronary artery disease, atrial fibrillation, chronic kidney disease, long-term dialysis, chronic obstructive pulmonary disease, and malignant tumors. The Charlson Comorbidity Index was also calculated to assess overall disease burden. Event history included MI, heart failure, and ischemic stroke requiring hospitalization. Medications were categorized as antithrombotic agents, glucose-lowering drugs, and other medications (primarily antihypertensive and lipid-lowering agents).

### Statistical Analysis

#### Primary Analysis

To minimize selection bias and confounding, inverse probability of treatment weighting (IPTW) was applied to create a weighted cohort based on the average treatment effect. Many studies have reported that propensity scores estimated using machine learning methods (such as a gradient boosting machine) often outperform those derived from traditional statistical models (such as logistic regression).^23^ Propensity scores were estimated using a gradient boosting machine algorithm (10 000 trees), regressing the study group (GLP-1 RAs vs DPP-4 inhibitors) on all baseline covariates. The index date was also carefully considered to ensure a comparable starting point for both groups, thereby allowing similar follow-up durations under the assumption of no difference in mortality between the groups. Covariate balance before and after weighting was assessed using absolute standardized differences (STDs), with an STD < 0.1 indicating good balance.

To assess the relative risk of fatal outcomes (including all-cause mortality, cardiovascular death, and MACEs) between the 2 groups, Cox proportional hazards models were employed. For nonfatal outcomes such as MALEs, the Fine and Gray subdistribution hazards model was used to account for the competing risk of death. Both the Cox proportional hazards model and Fine and Gray subdistribution hazards model rely on the assumption of proportional hazards. To ensure the robustness and reproducibility of our findings, we also performed restricted mean survival time analysis, which does not require the proportional hazards assumption and therefore provides a complementary assumption-free assessment of the treatment effect. The study group was the only explanatory variable in the aforementioned regression models. A 2-sided *P* < .05 was considered statistically significant. All statistical analyses were conducted using SAS version 9.4 (SAS Institute).

#### Sensitivity Analysis

In the primary analysis, discontinuation of the study drugs was not treated as a censoring event. Therefore, we conducted a sensitivity analysis in which treatment discontinuation was also considered as one of the censoring conditions. Moreover, because the primary analysis applied a 90-day grace period, we further checked robustness by extending the grace period to 180 days. This approach resulted in the exclusion of a larger number of patients and required repeating the IPTW procedure and outcome analyses to reassess the consistency of the findings. Finally, we conducted subgroup analyses of the major lower limb outcomes stratified by history of major amputation.

## Results

### Baseline Characteristics

A total of 19 676 patients were initially identified, of whom 2388 (12.1%) were excluded after applying the exclusion criteria (Figure 1). Of the 17 288 patients (mean [SD] age, 70.7 [12.0] years; 10 010 male [57.9%]) eligible for analysis, 1583 received GLP-1 RAs (liraglutide: 590 patients; dulaglutide: 981 patients; semaglutide: 12 patients), and 15 705 received DPP-4 inhibitors. Of all patients, 8535 (weighted percentage, 54.2.%) had revascularization, 8063 (weighted percentage, 56.9%) had CLTI, and 3264 (weighted percentage, 25.3%) had an amputation (major or minor).

Before adjustment, GLP-1 RA users were younger but had longer diabetes duration and higher prevalence of microvascular complications, prior revascularization, and cardioprotective medication use, reflecting a higher cardiovascular risk (Table 1). After IPTW adjustment, age, sex, microvascular complications, cardiovascular comorbidities, and medication use were well balanced, with STDs less than 0.1. The only residual imbalance was mean (SD) diabetes duration, which remained longer in the GLP-1 RA group (13.2 [5.2] vs 11.9 [5.7] years).

**Table 1.** Baseline Characteristics of Patients With Diabetes Who Received GLP1-RA vs DPP4i Therapy

Variable	Before weighting			After weighting		
	GLP1-RA, No. (%) (n = 1583)	DPP4i, No. (%) (n = 15 705)	Standardized difference (95% CI)	GLP1-RA, weighted % (n = 10 731.3)	DPP4i, weighted % (n = 17 072.0)	Standardized difference (95% CI)
Age, mean (SD), year	65.2 (12.2)	71.3 (11.8)	−0.51 (−0.56 to −0.45)	68.9 (11.6)	70.8 (11.9)	−0.17 (−0.19 to −0.14)
Sex						
Male	1002 (63.3)	9008 (57.4)	0.12 (0.07 to 0.17)	62.8	57.9	0.10 (0.08 to 0.13)
Female	581 (36.7)	6697 (42.6)	−0.12 (−0.17 to −0.07)	37.2	42.1	−0.10 (−0.13 to −0.08)
Diabetes duration, mean (SD), year	16.0 (5.3)	11.6 (5.6)	0.82 (0.76 to 0.87)	13.2 (5.2)	11.9 (5.7)	0.23 (0.20 to 0.25)
Diabetes duration grouping, y						
<10	228 (14.4)	4945 (31.5)	−0.42 (−0.47 to −0.36)	22.8	30.2	−0.17 (−0.19 to −0.14)
≥10	1355 (85.6)	10 760 (68.5)	0.42 (0.36 to 0.47)	77.2	69.8	0.17 (0.14 to 0.19)
Diabetes complications						
Retinopathy	333 (21.0)	1795 (11.4)	0.26 (0.21 to 0.31)	14.4	12.2	0.07 (0.04 to 0.09)
Autonomic or peripheral neuropathy	719 (45.4)	3910 (24.9)	0.44 (0.39 to 0.49)	33.1	26.5	0.14 (0.12 to 0.17)
Prior lower extremity arterial disease						
Amputation						
Minor	176 (11.1)	1544 (9.8)	0.04 (−0.01 to 0.09)	9.9	10.0	0.00 (−0.03 to 0.02)
Major	84 (5.3)	1460 (9.3)	−0.15 (−0.21 to −0.10)	6.7	9.0	−0.09 (−0.11 to −0.06)
Revascularization (EVT or bypass)	970 (61.3)	7565 (48.2)	0.27 (0.21 to 0.32)	52.1	49.1	0.06 (0.04 to 0.08)
Chronic limb-threatening ischemia	800 (50.5)	7263 (46.3)	0.09 (0.03 to 0.14)	46.1	46.5	−0.01 (−0.03 to 0.02)
Comorbid conditions						
Hypertension	1290 (81.5)	12 312 (78.4)	0.08 (0.03 to 0.13)	80.3	78.6	0.04 (0.02 to 0.07)
Dyslipidemia	1031 (65.1)	7219 (46.0)	0.39 (0.34 to 0.44)	54.3	47.5	0.14 (0.11 to 0.16)
Coronary artery disease	325 (20.5)	4107 (26.2)	−0.13 (−0.18 to −0.08)	21.9	25.7	−0.09 (−0.11 to −0.06)
Atrial fibrillation	112 (7.1)	1184 (7.5)	−0.02 (−0.07 to 0.03)	8.2	7.5	0.02 (0.00 to 0.05)
Chronic kidney disease	1073 (67.8)	7908 (50.4)	0.36 (0.31 to 0.41)	57.8	51.6	0.12 (0.10 to 0.15)
Long-term dialysis	326 (20.6)	2333 (14.9)	0.15 (0.10 to 0.20)	17.2	15.3	0.05 (0.03 to 0.08)
Chronic obstructive pulmonary disease	161 (10.2)	1509 (9.6)	0.02 (−0.03 to 0.07)	9.8	9.6	0.01 (−0.02 to 0.03)
Malignant tumor	102 (6.4)	1422 (9.1)	−0.10 (−0.15 to −0.05)	9.4	8.9	0.02 (−0.01 to 0.04)
CCI score						
Mean (SD)	3.9 (1.9)	3.6 (2.0)	0.18 (0.13 to 0.23)	3.8 (2.1)	3.6 (2.0)	0.11 (0.08 to 0.13)
Median (IQR)	4 (2 to 5)	3 (2 to 5)	NA	4 (2 to 5)	3 (2 to 5)	NA
CCI score grouping						
0-1	132 (8.3)	2204 (14.0)	−0.18 (−0.23 to −0.13)	10.8	13.6	−0.09 (−0.11 to −0.06)
2-3	584 (36.9)	6246 (39.8)	−0.06 (−0.11 to −0.01)	38.9	39.5	−0.01 (−0.04 to 0.01)
4-5	550 (34.7)	4726 (30.1)	0.10 (0.05 to 0.15)	31.9	30.4	0.03 (0.01 to 0.06)
≥6	317 (20.0)	2529 (16.1)	0.10 (0.05 to 0.15)	18.4	16.4	0.05 (0.03 to 0.08)
History of events						
Myocardial infarction	294 (18.6)	2064 (13.1)	0.15 (0.10 to 0.20)	13.9	13.5	0.01 (−0.01 to 0.03)
Heart failure	454 (28.7)	3760 (23.9)	0.11 (0.06 to 0.16)	26.9	24.3	0.06 (0.04 to 0.08)
Ischemic stroke	336 (21.2)	4122 (26.3)	−0.12 (−0.17 to −0.07)	21.9	25.9	−0.09 (−0.12 to −0.07)
Concomitant antithrombotic agents						
Aspirin	768 (48.5)	7027 (44.7)	0.08 (0.02 to 0.13)	43.4	45.0	−0.03 (−0.06 to −0.01)
Clopidogrel	549 (34.7)	3791 (24.1)	0.23 (0.18 to 0.28)	27.0	24.8	0.05 (0.03 to 0.07)
Ticagrelor or prasugrel	68 (4.3)	230 (1.5)	0.17 (0.12 to 0.22)	2.6	1.6	0.07 (0.05 to 0.09)
Cilostazol	573 (36.2)	5883 (37.5)	−0.03 (−0.08 to 0.03)	33.4	37.3	−0.08 (−0.11 to −0.06)
Oral anticoagulants	165 (10.4)	1425 (9.1)	0.05 (−0.01 to 0.10)	10.4	9.2	0.04 (0.02 to 0.07)
Concomitant glucose-lowering drugs						
Metformin	795 (50.2)	9029 (57.5)	−0.15 (−0.20 to −0.09)	55.4	57.0	−0.03 (−0.06 to −0.01)
Sulfonylurea	760 (48.0)	7241 (46.1)	0.04 (−0.01 to 0.09)	47.8	46.1	0.03 (0.01 to 0.06)
Thiazolidinedione	344 (21.7)	1537 (9.8)	0.33 (0.28 to 0.38)	14.0	10.7	0.10 (0.08 to 0.13)
α-Glucosidase inhibitors	228 (14.4)	2345 (14.9)	−0.01 (−0.07 to 0.04)	17.3	14.9	0.07 (0.04 to 0.09)
Nonsulfonylurea insulin secretagogues	314 (19.8)	2741 (17.5)	0.06 (0.01 to 0.11)	19.4	17.6	0.05 (0.02 to 0.07)
SGLT2i	357 (22.6)	718 (4.6)	0.54 (0.49 to 0.60)	8.3	5.9	0.09 (0.07 to 0.11)
Insulin	665 (42.0)	3866 (24.6)	0.38 (0.32 to 0.43)	28.6	25.9	0.06 (0.04 to 0.08)
Other concomitant medications						
RASi	694 (43.8)	7459 (47.5)	−0.07 (−0.13 to −0.02)	43.1	47.3	−0.08 (−0.11 to −0.06)
β-Blockers	893 (56.4)	6904 (44.0)	0.25 (0.20 to 0.30)	47.9	44.8	0.06 (0.04 to 0.09)
DCCBs	522 (33.0)	6524 (41.5)	−0.18 (−0.23 to −0.13)	36.0	41.0	−0.10 (−0.13 to −0.08)
Statins	1159 (73.2)	8134 (51.8)	0.45 (0.40 to 0.51)	61.8	53.5	0.17 (0.14 to 0.19)
Ezetimibe	207 (13.1)	849 (5.4)	0.27 (0.22 to 0.32)	7.4	5.9	0.06 (0.03 to 0.08)
NSAIDs or COX-2 inhibitors	794 (50.2)	8226 (52.4)	−0.04 (−0.10 to 0.01)	52.2	52.3	0.00 (−0.02 to 0.02)
Diuretics	424 (26.8)	4291 (27.3)	−0.01 (−0.06 to 0.04)	23.9	27.3	−0.08 (−0.10 to −0.05)
Spironolactone	162 (10.2)	1370 (8.7)	0.05 (0.00 to 0.10)	8.1	8.8	−0.03 (−0.05 to 0.00)
Follow up, mean (SD), y	3.2 (2.1)	4.2 (3.1)	−0.36 (−0.41 to −0.31)	3.6 (2.5)	4.1 (3.1)	−0.16 (−0.19 to −0.14)

Abbreviations: CCI, Charlson Comorbidity Index; COX-2, cyclooxygenase-2; DCCBs, dihydropyridine calcium channel blockers; DPP4i, dipeptidyl peptidase-4 inhibitor; EVT, endovascular therapy; GLP1-RA, glucagon-like peptide-1 receptor agonist; NSAID, nonsteroidal anti-inflammatory drug; RASi, renin-angiotensin-system inhibitor; SGLT2i, sodium-glucose cotransporter 2 inhibitor.

### Clinical Outcomes

**Figure 2.**
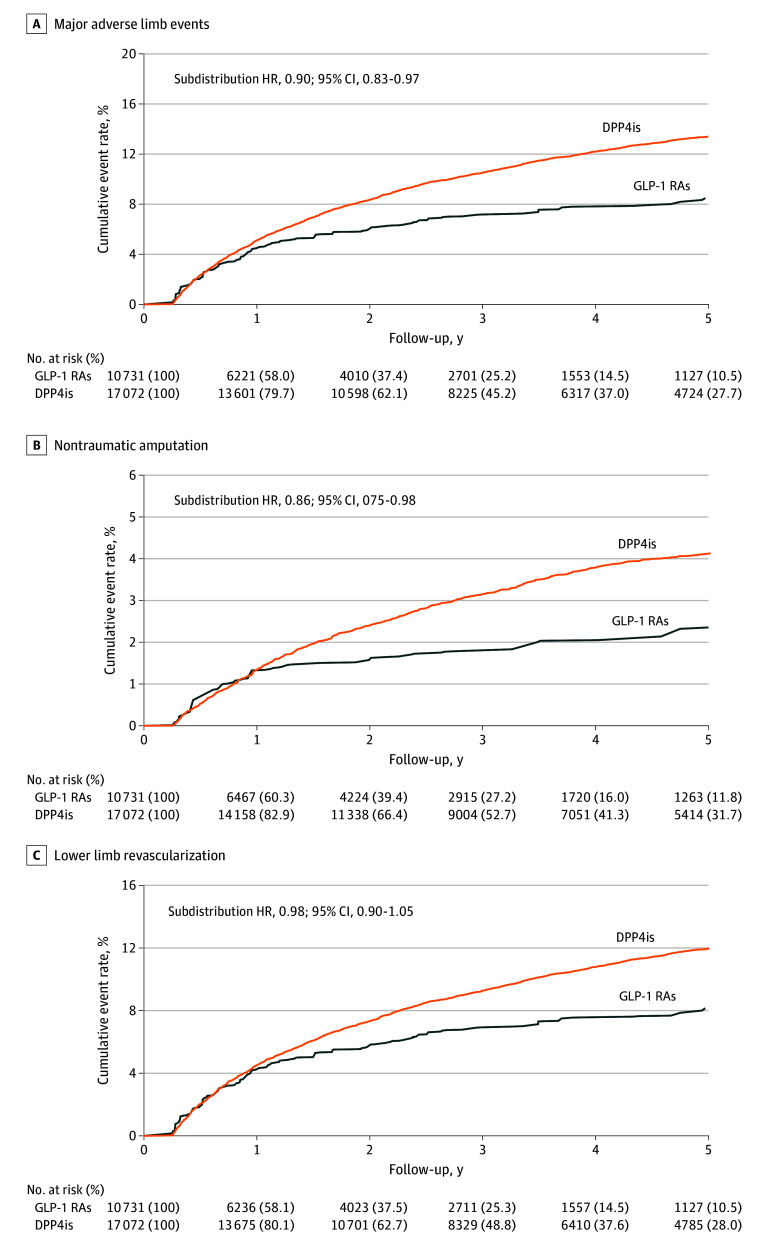
Cumulative Event Rates of Major Adverse Limb Events, Nontraumatic Amputations, and Lower Limb Revascularization Among Patients Who Received Glucagon-Like Peptide-1 Receptor Agonists (GLP-1 RAs) vs Dipeptidyl Peptidase-4 Inhibitors (DPP4is) in the Inverse Probability of Treatment Weighting–Adjusted Cohort HR indicates hazard ratio.

The overall mean (SD) follow-up duration was 4.1 (3.0) years in the original cohort. The overall incidence of the composite outcome consisting of revascularization or amputation was 42.4 (95% CI, 39.7-45.1) events per 1000 person-years in the GLP-1 RA group and 42.7 (95% CI, 41.1-44.3) events per 1000 person-years in the DPP-4 inhibitor group, resulting in a lower risk of MALEs for the GLP-1 RA group (subdistribution hazard ratio [SHR], 0.90; 95% CI, 0.83-0.97) (Table 2 and Figure 2A). The observed benefit in the primary composite outcome was primarily attributed to a significantly lower risk of overall and major amputation among the GLP-1 RA users. The incidence of overall amputation was 12.0 (95% CI, 7.2-9.6) events per 1000 person-years in the GLP-1 RA group, compared with 13.2 (95% CI, 12.4-14.1) events per 1000 person-years in the DPP-4 inhibitor group (SHR, 0.86; 95% CI, 0.75-0.98). This reduction was due to a marked decrease in the risk of major amputation, with an incidence of 4.4 (95% CI, 3.6-5.3) events per 1000 person-years in the GLP-1 RA group vs 7.2 (95% CI, 6.6-7.8) events per 1000 person-years in the DPP-4 inhibitor group (SHR, 0.59; 95% CI, 0.47-0.73) (Figure 2B). The incidence of revascularization was similar between the 2 groups, at 40.7 (95% CI, 38.0-43.4) events per 1000 person-years in the GLP-1 RA group and 37.9 (95% CI, 36.4-39.4) events per 1000 person-years in the DPP-4 inhibitor group (Figure 2C).

**Table 2.** Clinical Events of the Diabetic Patients Who Received GLP1-RA vs DPP4i Therapy in the Inverse Probability of Treatment Weighting–Adjusted Cohort

Outcome	Incidence rate (95% CI)^a^		GLP1-RA outcome risk, HR (95% CI)	*P* value	Change in restricted mean survival time, mo (95% CI)
	GLP1-RA (n = 10 731.3)	DPP4i (n = 17 072.0)			
Primary composite outcome^b^	42.4 (39.7-45.1)	42.7 (41.1-44.3)	0.90 (0.83-0.97)^c^	.005	5.9 (1.1-11.3)
Component of primary outcome					
Lower limb revascularization	40.7 (38.0-43.4)	37.9 (36.4-39.4)	0.98 (0.90-1.05)^c^	.53	7.2 (2.1-12.5)
Amputation	12.0 (10.6-13.4)	13.2 (12.4-14.1)	0.86 (0.75-0.98)^c^	.03	6.4 (2.3-12.0)
Minor	8.4 (7.2-9.6)	7.5 (6.9-8.2)	1.05 (0.89-1.24)^c^	.54	7.5 (4.0-12.6)
Major	4.4 (3.6-5.3)	7.2 (6.6-7.8)	0.59 (0.47-0.73)^c^	<.001	4.5 (1.9-9.2)
Secondary outcome					
Cardiovascular death	45.3 (42.6-48.1)	80.3 (78.2-82.4)	0.57 (0.53-0.61)	<.001	11.4 (4.8-17.5)
Ischemic stroke	13.7 (12.2-15.2)	15.9 (14.9-16.9)	0.79 (0.69-0.89)^c^	<.001	5.2 (0.1-11.5)
Acute myocardial infarction	10.5 (9.2-11.8)	18.3 (17.3-19.3)	0.53 (0.46-0.60)^c^	<.001	1.6 (1.4-6.2)
Major adverse cardiovascular events^d^	65.8 (62.4-69.1)	103.1 (100.6-105.5)	0.62 (0.58-0.65)	<.001	9.1 (2.3-15.7)
All-cause death	90.2 (86.3-94.0)	143.7 (140.9-146.5)	0.63 (0.60-0.66)	<.001	41.2 (36.3-45.5)
New onset of long-term dialysis	13.4 (11.9-14.9)	21.6 (20.5-22.7)	0.61 (0.54-0.70)^c^	<.001	0.7 (2.7-5.4)

Abbreviations: DDP4i, dipeptidyl peptidase-4 inhibitor; GLP-1 RA, glucagon-like peptide-1 receptor agonist; HR, hazard ratio.

^a^ Number of events per 1000 person-years.

^b^ Lower limb revascularization or amputation.

^c^ Subdistribution HR.

^d^ Cardiovascular death, ischemic stroke, or acute myocardial infarction.

**Figure 3.**
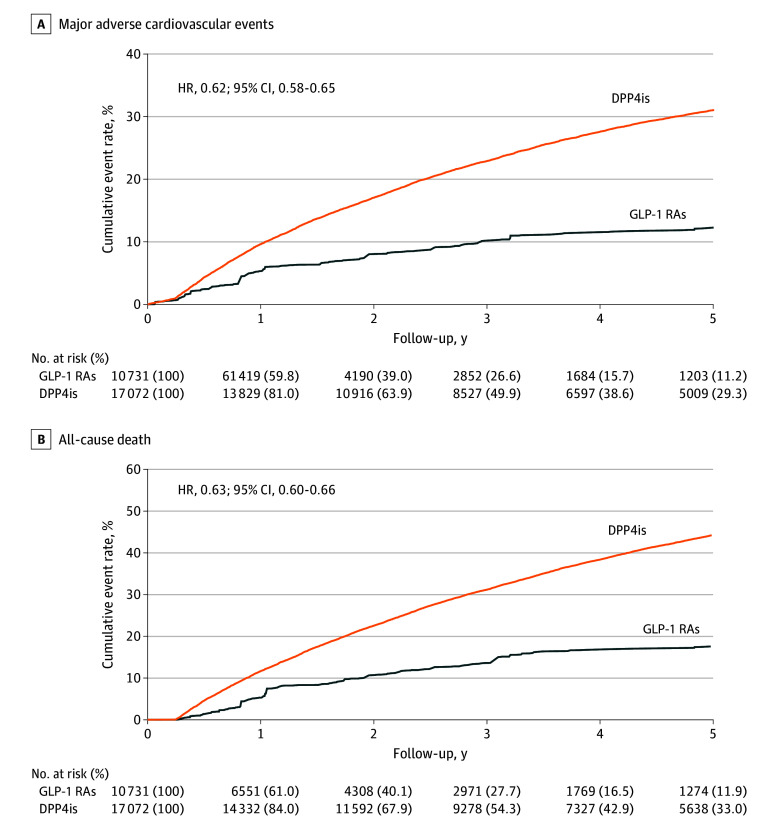
Cumulative Event Rates of Major Adverse Cardiovascular Events and All-Cause Death Among the Patients Who Received Glucagon-Like Peptide-1 Receptor Agonists (GLP-1 RAs) vs Dipeptidyl Peptidase-4 Inhibitors (DPP4is) in the Inverse Probability of Treatment Weighting–Adjusted Cohort HR indicates hazard ratio.

The incidence of MACEs was significantly lower among the GLP-1 RA users compared with the DPP-4 inhibitor users (65.8 [95% CI, 62.4-69.1] events per 1000 person-years vs 103.1 [95% CI, 100.6-105.5] events per 1000 person-years; HR, 0.62, 95% CI, 0.58-0.65) (Figure 3A). The incidence of each individual MACE component was also lower in the GLP-1 RA group compared with the DPP-4 inhibitor group (cardiovascular death: 45.3 [95% CI, 42.6-48.1] events per 1000 person-years vs 80.3 [95% CI, 78.2-82.4] events per 1000 person-years; HR 0.57; 95% CI, 0.53-0.61; ischemic stroke: 13.7 [95% CI, 12.2-15.2] events per 1000 person-years vs 15.9 [95% CI, 14.9-16.9] events per 1000 person-years; SHR 0.79; 95% CI, 0.69-0.89); acute MI: 10.5 [95% CI, 9.2-11.8] events per 1000 person-years vs 18.3 [95% CI, 17.3-19.3] events per 1000 person-years; SHR 0.53; 95% CI, 0.46-0.60). In addition, all-cause mortality was significantly lower in the GLP-1 RA group (90.2 [95% CI, 86.3-94.0] events per 1000 person-years) compared with the DPP-4 inhibitor group (143.7 [95% CI, 140.9-146.5] events per 1000 person-years) (HR, 0.63; 95% CI, 0.60-0.66) (Figure 3B). The incidence of progression to long-term dialysis was also significantly lower in the GLP-1 RA group compared with the DPP-4 inhibitor group (13.4 [95% CI, 11.9-14.9] events per 1000 person-years vs 21.6 [95% CI, 20.5-22.7] events per 1000 person-years; SHR 0.61, 95% CI, 0.54-0.70). The restricted mean survival time analysis yielded even more pronounced differences between the 2 groups, with the outcomes of lower limb revascularization and minor amputation that were not statistically significant in the Cox or Fine and Gray models becoming significant in the restricted mean survival time analysis (Table 2).

### Sensitivity Analysis

The results obtained when also treating discontinuation of the study drugs as a censoring event were highly consistent with those of the primary analysis; the GLP-1 RA group continued to have significantly lower risks of the primary composite lower-limb outcome, major amputation, and cardiovascular outcomes (eTable 1 in Supplement 1). Moreover, extending the grace period from 90 to 180 days yielded findings that were even more favorable for the GLP-1 RA group, with lower-limb revascularization also reaching statistical significance (eTable 2 in Supplement 1). Subgroup analysis of the major lower limb outcome stratified by prior major amputation demonstrated that the beneficial outcome observed in the GLP-1 RA group was attenuated among the patients with a previous history of major amputation (eTable 3 in Supplement 1).

## Discussion

The main finding of this cohort study was that the patients with diabetes and a history of MALEs who received GLP-1 RAs had lower risks of MALEs, amputation, MACEs, all-cause death, and progression to long-term dialysis compared with those who received DPP-4 inhibitors. To our knowledge, this is the first study to evaluate the comparative efficacy outcomes of glucose-lowering therapies for secondary MALE prevention in a high-risk cohort characterized by established revascularization (54.2%), CLTI (56.9%), and prior amputation (25.3%). In contrast, previous investigations focused on populations with significantly lower baseline risk. Lin et al^24^ and Baviera et al^25^ observed reduced limb events with GLP-1 RAs in patients with type 2 diabetes, yet their cohorts had minimal histories of severe limb complications (0.1%-1.0%). Similarly, our prior multicenter analysis^26^ and the study by Schafer et al^27^ reported protective associations in groups with low baseline LEAD prevalence (2.1%-6.5%). Data from the Danish National Health Service^28^ further corroborate these trends in lower-risk populations. Taken together, these findings and ours suggest the consistent benefits of GLP-1 RAs in reducing the risk of adverse limb outcomes across diverse patient populations, potentially in both primary prevention (in lower-risk individuals) and secondary prevention (in higher-risk individuals such as in the present study).

Patients with a history of MALEs have extensive polyvascular atherosclerosis and substantial comorbidities, leading to markedly elevated cardiovascular risk. In the Cardiovascular Outcomes for People Using Anticoagulation Strategies trial, MALEs were associated with markedly higher subsequent mortality (HR, 3.23; 95% CI, 1.87-5.56).^2^ Likewise, a large database study^4^ reported that hospitalization for a MALE after revascularization significantly increased the risk of subsequent MI or stroke (HR, 1.34; 95% CI 1.28-1.40), with risk increasing over time. In this context, our finding that GLP-1 RA use was associated with lower MACEs, cardiovascular mortality, and all-cause mortality is consistent with major cardiovascular outcome trials.^29,30,31^ Evidence suggests these benefits may be greater among higher-risk patients; a recent methodological framework for cardiovascular outcome trial meta-analysis^32^ showed significantly larger absolute risk reductions in MACEs among patients with prior cardiovascular disease treated with GLP-1 RAs. Similarly, a related meta-analysis^33^ reported a much lower number needed to treat for secondary prevention (number needed to treat = 21) than for primary prevention (number needed to treat = 82). Collectively, these data support prioritizing GLP-1 RAs for patients with established cardiovascular disease—including those with prior MALEs—to maximize clinical benefit.

More than one-half of participants in our study had chronic kidney disease, and the reduced progression to dialysis aligns with prior evidence. In the trial by Perkovic et al,^34^ semaglutide lowered the risk of kidney disease progression by 24% and cardiovascular mortality by 29% in patients with type 2 diabetes and chronic kidney disease. Similarly, a recent meta-analysis of more than 85 000 participants across 11 large trials reported that GLP-1 RAs reduced kidney failure by 16%, slowed kidney function decline by 22%, and decreased kidney disease–related mortality by approximately 19%.^35^

In this study, treatment with GLP-1 RAs was associated with significantly lower risks of MACEs and MALEs in patients with diabetes and a history of MALEs. The observed benefits likely stem from synergistic systemic and direct vascular mechanisms. Systemically, GLP-1 RAs improve glycemic control, weight, blood pressure, and lipid profiles while attenuating inflammation. Direct vasculoprotection is mediated via vascular GLP-1 receptors, which enhance endothelial function, reduce oxidative stress, and inhibit smooth muscle cell proliferation. These antiatherogenic actions limit plaque progression, macrophage activation, and foam-cell formation. Furthermore, the preservation of endothelial integrity and promotion of angiogenesis provide a coherent biological basis for the reduced cardiovascular and limb events observed in this high-risk population.^36,37,38^

While GLP-1 RAs reduced major amputation and MACE, revascularization rates remained unchanged. This discordance likely reflects the heterogeneity of revascularization, which encompasses both urgent limb salvage and elective interventions influenced by clinician preference, symptom perception, and health care access. In contrast, major amputation and MACE represent definitive, high-severity events, primarily due to biological disease progression rather than elective decision-making. Consequently, the reduction in these hard end points suggests a true biological benefit of GLP-1 RAs, whereas revascularization rates may be confounded by nonbiological variation in clinical practice.

Subgroup analysis revealed attenuated protection against recurrent amputation in patients with prior major limb loss. However, this subgroup comprised only 8.9% of the cohort, representing a population with irreversible pathology and advanced disease. These findings should be viewed as hypothesis-generating, potentially reflecting limited statistical power, unmeasured frailty, or residual confounding. Additionally, GLP-1 RA–induced weight loss might compromise the nutritional reserves necessary for healing in these vulnerable patients. Thus, while GLP-1 RAs appear robust for preventing initial major amputations, their efficacy in patients with established major limb loss remains uncertain and warrants prospective investigation.

### Limitations

This study has limitations inherent to its observational design. First, despite using IPTW to balance baseline characteristics, residual confounding may persist. Data on socioeconomic status, lifestyle factors (eg, smoking), and laboratory values (glycohemoglobin, cholesterol, creatinine, and urine albumin creatinine ratio) were unavailable and could influence outcomes. Second, information on ankle-brachial index, wound severity, and limb perfusion was lacking, limiting the assessment of LEAD severity. Third, the smaller sample size of the GLP-1 RA group compared with the DPP-4 inhibitor group may have introduced selection bias because GLP-1 RA users were younger and potentially more engaged with health care services. Fourth, generalizability may be limited by Taiwan’s reimbursement policies, which restrict the concomitant use of GLP-1 RAs and sodium-glucose cotransporter 2 inhibitors. Fifth, we could not distinguish whether recurrent MALE occurred in the ipsilateral or contralateral limb because laterality was not consistently coded. Sixth, the observational nature of the study precludes the establishment of a causal association, and prospective randomized clinical trials are needed to confirm the benefits of GLP-1 RAs in reducing MALEs and cardiovascular outcomes in this high-risk population.

## Conclusions

In this nationwide cohort study of patients with diabetes and prior MALEs, treatment with GLP-1 RAs was associated with significantly lower risks of recurrent limb events, cardiovascular events, all-cause mortality, and kidney disease progression compared with DPP-4 inhibitors. These findings support the preferential use of GLP-1 RAs for secondary prevention in this high-risk population.

## Notes

Supplement 1. eTable 1. Clinical events of the diabetic patients who received GLP1-RAs versus DPP4i therapy in the IPTW-adjusted cohort, additionally treating discontinuation of the study drugs as a censoring event eTable 2. Clinical events of the diabetic patients who received GLP1-RAs versus DPP4i therapy in the IPTW-adjusted cohort, with the grace period of 180 days eTable 3. Subgroup analysis of major lower limb outcome by the presence or absence of previous history of major amputation

Supplement 2. Data Sharing Statement
