# Supplementary material for: Glucagon-Like Peptide-1 Receptor Agonists and Prior Major Adverse Limb Events in Patients With Diabetes
Source: JAMA Netw Open. 2026 Jan 28;9(1):e2555952. doi: 10.1001/jamanetworkopen.2025.55952 (PMC12853205; doi:10.1001/jamanetworkopen.2025.55952)
Supplement: Supplement 1. — eTable 1. Clinical events of the diabetic patients who received GLP1-RAs versus DPP4i therapy in the IPTW-adjusted cohort, additionally treating discontinuation of the study drugs as a censoring event eTable 2. Clinical events of the diabetic patients who received GLP1-RAs versus DPP4i therapy in the IPTW-adjusted cohort, with the grace period of 180 days eTable 3. Subgroup analysis of major lower limb outcome by the presence or absence of previous history of major amputation [file jamanetwopen-e2555952-s001.pdf]

Supplemental Online Content

Hsiao F-C, Hsu T-J, Hsieh Y-J, et al. Glucagon-like peptide-1 receptor agonists and prior major adverse limb events in patients with diabetes. *JAMA Netw. Open*. 2026;9(1):e2555952. doi: 10.1001/jamanetworkopen.2025.55952

eTable 1. Clinical events of the diabetic patients who received GLP1-RAs versus DPP4i therapy in the IPTW-adjusted cohort, additionally treating discontinuation of the study drugs as a censoring event

eTable 2. Clinical events of the diabetic patients who received GLP1-RAs versus DPP4i therapy in the IPTW-adjusted cohort, with the grace period of 180 days

eTable 3. Subgroup analysis of major lower limb outcome by the presence or absence of previous history of major amputation

This supplemental material has been provided by the authors to give readers additional information about their work.

© 2025 Hsiao F-C et al. *JAMA Network Open*.

eTable 1. Clinical events of the diabetic patients who received GLP1-RAs versus DPP4i therapy in the IPTW-adjusted cohort, additionally treating discontinuation of the study drugs as a censoring event

	Incidence rate* (95% CI)			
Outcome	GLP1-RAs (*n* = 10,731.3)	DPP4i (*n* = 17,072.0)	HR/SHR for GLP1-RA (95% CI)	*P* value
Primary composite outcome#	42.5 (39.7–45.3)	42.7 (41.1–44.3)	0.90 (0.84–0.97)	0.007
Component of primary outcome				
Lower limb revascularization	40.9 (38.2–43.6)	37.9 (36.4–39.4)	0.98 (0.91–1.06)	0.618
Amputation	11.9 (10.5–13.3)	13.2 (12.4–14.1)	0.85 (0.74–0.97)	0.020
Minor	8.3 (7.1–9.5)	7.5 (6.9–8.2)	1.04 (0.88–1.23)	0.654
Major	4.4 (3.5–5.2)	7.2 (6.6–7.8)	0.58 (0.46–0.72)	<0.001
Secondary outcome				
Cardiovascular death	45.6 (42.8–48.3)	80.3 (78.2–82.4)	0.57 (0.54–0.61)	<0.001
Ischemic stroke	13.8 (12.2–15.3)	15.9 (14.9–16.9)	0.79 (0.70–0.90)	<0.001
Acute myocardial infarction	10.6 (9.2–11.9)	18.3 (17.3–19.3)	0.53 (0.46–0.61)	<0.001
Major adverse cardiovascular events†	66.1 (62.8–69.5)	103.1 (100.6–105.5)	0.62 (0.59–0.66)	<0.001
All-cause death	90.6 (86.7–94.5)	143.7 (140.9–146.5)	0.63 (0.60–0.66)	<0.001
New onset of chronic dialysis	13.4 (11.9–14.9)	21.6 (20.5–22.7)	0.62 (0.54–0.70)	<0.001

Abbreviation: IPTW, inverse probability of treatment weighting; DDP4i, dipeptidyl peptidase-4 inhibitors; GLP-1 RAs, glucagon-like peptide-1 receptor agonists; CI, confidence interval; SHR, subdistribution hazard ratio; ID, incidence density; HR, hazard ratio; SHR, subdistribution hazard ratio; MACE, major cardiovascular events;

* Number of events per 1,000 person-years;

# Anyone of lower limb revascularization or amputation;

† Anyone of cardiovascular death, ischemic stroke and acute myocardial infarction.

© 2025 Hsiao F-C et al. *JAMA Network Open*.

eTable 2. Clinical events of the diabetic patients who received GLP1-RAs versus DPP4i therapy in the IPTW-adjusted cohort, with the grace period of 180 days

	Incidence rate* (95% CI)			
Outcome	GLP1-RAs (*n* = 10,731.3)	DPP4i (*n* = 17,072.0)	HR/SHR for GLP1-RA (95% CI)	*P* value
Primary composite outcome#	30.3 (27.8–32.8)	36.1 (34.6–37.6)	0.80 (0.73–0.87)	<0.001
Component of primary outcome				
Lower limb revascularization	29.5 (27.0–31.9)	32.3 (30.8–33.7)	0.87 (0.79–0.95)	0.003
Amputation	7.1 (5.9–8.2)	11.3 (10.5–12.1)	0.62 (0.52–0.75)	<0.001
Minor	5.5 (4.5–6.6)	6.6 (6.0–7.2)	0.82 (0.67–1.01)	0.065
Major	2.4 (1.7–3.0)	6.1 (5.5–6.7)	0.38 (0.28–0.52)	<0.001
Secondary outcome				
Cardiovascular death	43.8 (40.9–46.7)	73.6 (71.6–75.7)	0.63 (0.59–0.68)	<0.001
Ischemic stroke	13.2 (11.6–14.8)	15.8 (14.8–16.8)	0.76 (0.66–0.87)	<0.001
Acute myocardial infarction	7.3 (6.1–8.5)	17.4 (16.4–18.4)	0.39 (0.33–0.46)	<0.001
Major adverse cardiovascular events†	60.5 (57.0–63.9)	95.8 (93.3–98.2)	0.63 (0.60–0.68)	<0.001
All-cause death	84.6 (80.6–88.7)	131.5 (128.8–134.3)	0.68 (0.65–0.72)	<0.001
New onset of chronic dialysis	12.3 (10.8–13.9)	21.4 (20.3–22.5)	0.56 (0.49–0.64)	<0.001

Abbreviation: IPTW, inverse probability of treatment weighting; DDP4i, dipeptidyl peptidase-4 inhibitors; GLP-1 RAs, glucagon-like peptide-1 receptor agonists; CI, confidence interval; SHR, subdistribution hazard ratio; ID, incidence density; HR, hazard ratio; SHR, subdistribution hazard ratio; MACE, major cardiovascular events;

* Number of events per 1,000 person-years;

# Anyone of lower limb revascularization or amputation;

† Anyone of cardiovascular death, ischemic stroke and acute myocardial infarction.

© 2025 Hsiao F-C et al. *JAMA Network Open*.

eTable 3. Subgroup analysis of major lower limb outcome by the presence or absence of previous history of major amputation

Outcome / History of major amputation	GLP1-RAs (*n* = 10,731.3)	DPP4i (*n* = 17,072.0)	SHR for GLP1-RA (95% CI)	*P* for interaction
Minor amputation				0.141
No	8.4 (7.2–9.7)	7.5 (6.8–8.1)	1.06 (0.90–1.26)	
Yes	5.1 (0.8–9.5)	8.3 (5.8–10.7)	0.53 (0.22–1.32)	
Major amputation				<0.001
No	3.5 (2.7–4.3)	6.5 (5.9–7.2)	0.51 (0.40–0.65)	
Yes	22.8 (13.6–32.1)	15.5 (12.1–18.9)	1.31 (0.82–2.09)	
Any amputation				0.133
No	11.1 (9.7–12.5)	12.6 (11.7–13.5)	0.83 (0.72–0.96)	
Yes	28.0 (17.8–38.3)	21.1 (17.1–25.1)	1.16 (0.77–1.75)	

© 2025 Hsiao F-C et al. *JAMA Network Open*.
