# Supplementary material for: Glucagon-Like Peptide-1 Receptor Agonists and Prior Major Adverse Limb Events in Patients With Diabetes
Source: JAMA Netw Open. 2026 Jan 28;9(1):e2555952. doi: 10.1001/jamanetworkopen.2025.55952 (PMC12853205; doi:10.1001/jamanetworkopen.2025.55952)
Supplement: Supplement 2. — Data Sharing Statement [file jamanetwopen-e2555952-s002.pdf]

Data Sharing Statement

Hsiao. Glucagon-Like Peptide-1 Receptor Agonists and Prior Major Adverse Limb Events in Patients With Diabetes. *JAMA Netw Open*. Published January 28, 2026.
doi:10.1001/jamanetworkopen.2025.55952

Data
Data available: Yes
Data types: Deidentified participant data
How to access data: taipei.chu@gmal.com
When available: With publication

Supporting Documents
Document types: Informed consent form
How to access documents: taipei.chu@gmail.com
When available: With publication

Additional Information
Who can access the data: researchers whose proposed use of the data has been approved
Types of analyses: for study
Mechanisms of data availability: signed data access agreement
Any additional restrictions: CGMH IRB
